# My 50 Year Odyssey to Develop Behavioral Methods to Let Me See Quickly How Well Kittens See

**DOI:** 10.1523/ENEURO.0576-24.2025

**Published:** 2025-04-11

**Authors:** Donald E. Mitchell

**Affiliations:** Department of Psychology & Neuroscience, Dalhousie University, Halifax, NS B3H 4R2, Canada

**Keywords:** critical period, darkness, vision, visual acuity, visual deprivation

## Abstract

The importance of animal models to an understanding of the development and plasticity of visual functions was evident from the outset of the long experimental collaboration of David Hubel and Torsten Wiesel in the early 1960s. Their initial work on kittens had massive impact in part because of the recognition that kittens share with primates substantial similarities of visual system organization and plasticity (e.g., eye-specific lamination of the thalamus and columnar organization of the visual cortex), as well as comparable visual abilities (including stereoscopic vision). In addition the plasticity demonstrated in response to early periods of selected visual exposure provided a glimpse into the origins of amblyopia. Five decades ago my laboratory developed a method for the fast measurement of visual thresholds in kittens in order to capture the consequences for spatial vision of the rapid physiological changes that occurred in the visual cortex during both typical development and those that follow various forms of early selected visual exposure. This paper describes the further evolution of the method with an emphasis on the testing procedures that enable fast capture of spatial visual thresholds such as visual acuity on every animal and occasion. In these respects, the method emulated features of basic tests of human spatial vision as applied in clinical settings. As with clinical tests for humans, the method includes benchmarks of low vision necessary to document the profound immediate consequences of early selected visual deprivation and the speed and extent of the subsequent recovery.

## Significance Statement

The method was developed nearly 50 years ago in order to permit fast (∼30 min) and daily longitudinal measurement of various visual spatial thresholds such as visual acuity in individual kittens from ∼4 weeks of age to early adulthood. A key feature of the jumping stand and ancillary testing procedures is the ability to permit fast and longitudinal measurement of visual spatial thresholds in every kitten and occasion by use of procedures patterned on clinical as opposed to laboratory testing of humans.

## Introduction

In common with many others at the time, my early research career was guided by contemporary accounts of the drastic consequences of early monocular deprivation (MD) or squint for the visual cortex of kittens (Wiesel and Hubel, 1963, 1965; Hubel and Wiesel, 1965). These early reports and later demonstrations of the susceptibility of other stimulus preferences of cortical cells to early biased visual exposure (Blakemore and Cooper, 1970; Daw and Wyatt, 1976) provided insight into both experiential influences on the development of the visual cortex and plausible narratives for the origin of several forms of human amblyopia. However, as summarized in two engaging reviews (Movshon and Van Sluyters, 1981; Teller and Movshon, 1986) that describe the spectrum of views held at the time concerning experiential influences on the stimulus preferences of cortical neurons, disputes soon arose over the magnitude and nature of the cortical alterations induced by certain forms of early biased visual exposure. Division of opinion at the time was especially jarring over reports of the magnitude of experientially induced changes in the orientation selectivity of cortical neurons. As weighed against the multitude of anatomical and physiological investigations of the consequences of early biased visual exposure, exploration of the consequences for vision were sparse. The void created by the absence of behavioral data from visually deprived animals was addressed in part by psychophysical investigations of humans with either grossly abnormal early visual input as a result of early monocular or binocular cataracts (Lewis et al., 1995) or relatively minor disturbance of visual input as a consequence of high astigmatism that had not been optically corrected in childhood. As adults, optically corrected astigmats showed substantial residual deficits of acuity for certain orientations of gratings that had likely been seen as blurred prior to optical correction of their astigmatism (Freeman et al., 1972; Mitchell et al., 1973; Mitchell and Wilkinson, 1974). The results from astigmatic humans motivated my colleagues and I to turn to psychophysical studies of adult cats that as kittens had received biased visual exposure to a restricted range of contour orientations (the so-called stripe-rearing).

## Studies of Adult Animals after Early Postnatal Selected Visual Exposure

For our initial foray, Darwin Muir and I employed the cutting-edge operant procedures developed by Mark Berkley (Berkley, 1970; Berkley and Sprague, 1979) to document the visual deficits of stripe-reared cats that had as kittens been selectively exposed for several hours each day for several months to stripes of a single orientation. Prior to and following the daily period of exposure to stripes of one orientation (either vertical, horizontal, or diagonal), the kittens were kept in total darkness (Muir and Mitchell, 1973, 1975; Blasdel et al., 1977). Although leading edge at the time, the operant methods used to assess vision were slow and applicable only to mature animals, thereby allowing for possible recovery to occur from imposed early selected visual deprivation in the time before and after behavioral testing was initiated. As it was not possible to initiate training before 4 months of age and the subsequent measurement of visual thresholds required 3 or more months to complete, the animals were young adults upon completion of behavioral testing (Muir and Mitchell, 1973, 1975; Blasdel et al., 1977). The possibility that the deficits might decline following biased early visual exposure together with the urgency of the physiological changes observed in the visual cortex following short periods of MD (Hubel and Wiesel, 1970) provided the initial impetus for us to develop a new method that could capture the consequences for vision. In collaboration at the time with three graduate students, a technician, and a summer undergraduate student in late 1974 and the summer of 1975, my laboratory began a quest for a method that would allow longitudinal measurements of various spatial visual thresholds in young kittens, ideally on a daily basis.

## The Requirements for a Technique Applicable to Young Kittens

In the paper (Mitchell et al., 1976, p. 363) that materialized eventually from our venture to develop a method suitable for kittens, we stated that it was a “simple modification of the jumping stand developed by Lashley for rats (Lashley, 1930).” However, this statement reflects the blunt contemporary comments of a departmental colleague with expertise in animal learning upon being shown the method in the immediate aftermath of its development. Notwithstanding this prior history with rats the method emerged afresh at the end of a quest to meet the three requirements that it should be universal, i.e., allow frequent longitudinal measurement on every kitten; second, that it permit thresholds to be obtained fast; and third, allow measurement from 4 weeks of age until early adulthood. In aggregate these requirements directed our focus toward exploitation of patterns of natural motor behavior that emerged early at or ∼1 month of age that could provide a foundation for continued measurements of visual thresholds into adulthood. Our focus upon 1 month of age for initiation of behavioral measurement of spatial thresholds was dictated by its coincidence with maturation of the optical quality of the optical media (Bonds and Freeman, 1978), the age of maximum vulnerability of the visual cortex to MD (Hubel and Wiesel, 1970; Olson and Freeman, 1980; Mitchell and Maurer, 2022), and finally, the emergence of such basic motor activities as crawling, walking, and jumping. Our search for a method was guided by recognition that it was crucial that it rely on a simple motor activity that emerged spontaneously and early so that it could be exploited with minimal or no training. Finally, the profile of the critical period of vulnerability to MD in the visual cortex (Mitchell and Maurer, 2022) guided the further necessity that the method permit measurements of spatial visual thresholds until early adulthood (6–8 months).

For a short time we explored the use of a preferential looking (PL) procedure that had been developed earlier for measurement of spatial visual thresholds in human infants (Teller et al., 1974; Teller, 1997) and later for infant monkeys (Teller et al., 1978). Our experience with this method was negative due to a number of factors that included its labor-intensive nature in terms of the number of people required for its application, inconsistent results across measurements on the same and different kittens, and the impression that the method would not permit longitudinal assessment on individual kittens over a sufficiently long period of time. In retrospect our decision to abandon PL could be considered premature as subsequently other groups reported successful application of the method to study the development of visual acuity and texture discrimination in kittens (Sireteanu, 1985; Wilkinson, 1995).

**Figure 1.**
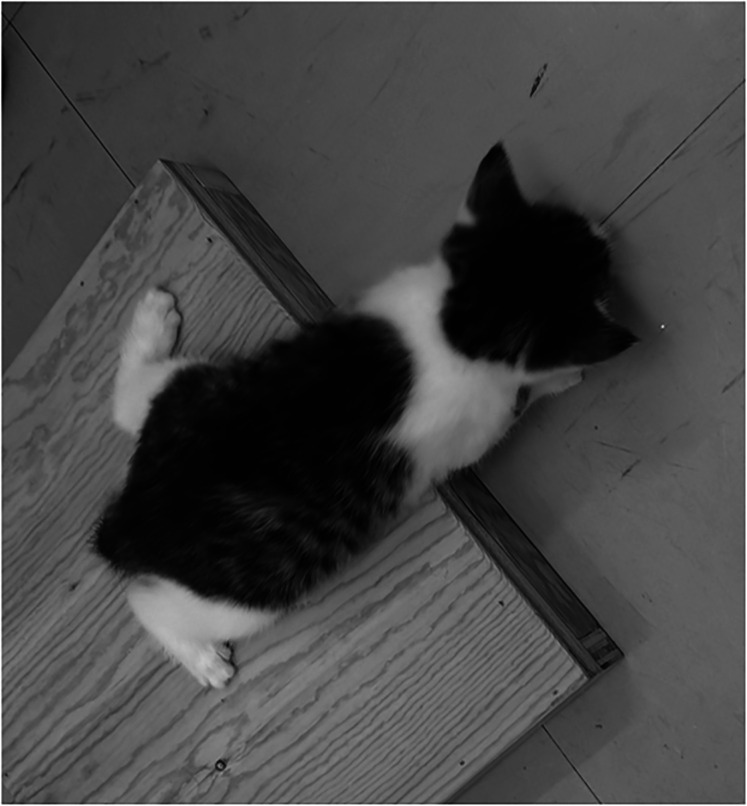
Photograph of a 4-week-old young kitten scrambling to the floor from a box only 2.9 cm high. The same kitten was unable to descend to the floor from the next highest box (5 cm high). This kitten was deemed too young to initiate training on the jumping stand on that day.

**Figure 2.**
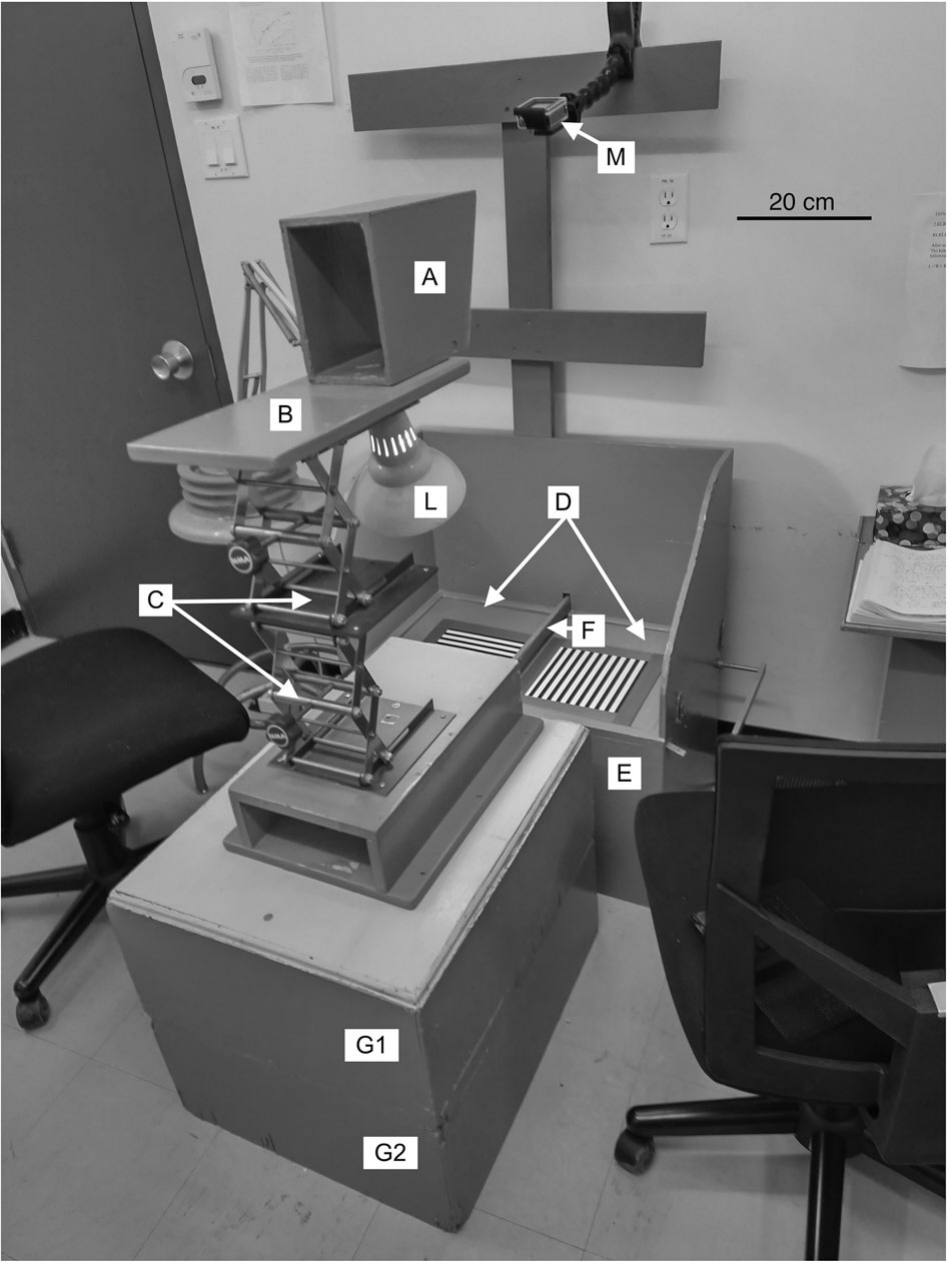
A photograph of an updated early model of the jumping stand where letters are used to designate key features. The scale bar is 20 cm long. A kitten is placed on the platform (B) and guided gently into the open-ended box (A) to jump toward the vertical grating below for a food reward accompanied by verbal praise and petting. The distance of the jumping platform to the stimuli can be adjusted in large steps by use of boxes of various sizes (G1 and G2) and in smaller continuous amounts by adjustment of the extension of two yoked laboratory jacks (C). If the kitten jumps instead to the adjacent horizontal grating (an error), it is placed immediately on the platform to repeat the trial without any reward. The gratings, illuminated by the lamp (L), are placed on two adjacent closed trapdoors (D) separated by a divider (F) that is 1 cm wide.

The next and ultimately successful step was to capitalize upon a natural disposition of kittens to initiate descents from an elevated surface. Following initial exploration of the extent of the surface upon which they are placed, kittens first peer down and then extend their front limbs in attempts to reach and scramble to the floor below. In the early stages of possible exploitation of this natural behavior, we constructed four boxes having identical surface dimensions (31 cm square) on top but of different heights (either 2.9, 5.0, 16.5, or 21.0 cm) that proved ultimately useful for selection of appropriate heights for the jumping platform prior to a testing session and for the initial assessment of the visual and motor abilities of young or selectively deprived kittens. As illustrated in the photograph of Figure 1, the eagerness of kittens to descend from elevated surfaces motivated us to explore whether jumps toward particular stimuli could be shaped by appropriate reinforcement, a search that proved finally successful in the form of a jumping stand that confronted kittens with a two-alternative forced-choice discrimination. As detailed descriptions of the apparatus were provided many years ago (Mitchell et al., 1976, 1977), it is possible here to summarize its key features by reference to a recent photograph of an upgraded version of the simplest design of a jumping stand built 40 years ago (Fig. 2). Photographic or laser printed stimuli with a firm backing were employed in the early jumping stands but for later versions, they were supplemented or supplanted by stimuli presented on computer monitors placed just underneath clear glass onto which the kittens jumped. Electronic modes of display were necessary for complex visual stimuli that incorporated attributes such as motion (Mitchell et al., 2009; Murphy et al., 2015). The original descriptions of the jumping stand did not provide a detailed account of the ancillary psychophysical test procedures that were devised in order to fulfill the important requirement that the method permit measurements to be made fast on every kitten and occasion. From the start we were guided by our experiences from use of prevailing operant methods and in particular with observations that acuities of 3 and 5 cycles/deg were critical levels for normal kittens to first attain and then exceed.

**Movie 1.** This movie illustrates the behavior of a kitten (C83) on the jumping stand on the last day of a period of monocular deprivation that began at P34 and ended at P71. Note that with its open eye, the kitten looks at both stimuli before it commits to a jump to the vertical grating (positive stimulus). [View online]

**Movie 2.** A very different pattern of behavior was evident for C83 the next day following minor surgery to open the closed eyelids of the deprived eye. To allow tests of the vision of the deprived eye, a hard opaque contact lens occluder was placed upon the cornea of the nondeprived eye preceded by a drop of a local ophthalmic anesthetic (Alcaine, proparacaine hydrochloride 0.5%). The movie clip opens with display of three different opaque contact lens occluders having different base curvatures alongside the local anesthetic. It appears that even when flush to the stimuli, the kitten was unable to detect the closed from an open trapdoor on the jumping stand by visual cues alone but instead had to resort to touch through extension of its front paw(s). This kitten was deemed to have insufficient vision to pass the “open door” test as it could only find the closed door on the basis of tactile information. [View online]

**Movie 3.** This movie illustrates rapid recovery of vision in a kitten (C166) following a 10 d period spent in total darkness from P37 to 47. Prior to that the kitten had received a period of monocular eyelid closure from P7 to P37 that had been terminated a few hours prior to being placed in a darkroom (Mitchell, 2013) for 10 d. A complete description of the recovery of the visual acuity in both eyes of C166 following its removal from the darkroom was published by Mitchell et al. (2016, their Fig. 1*A*). On first exposure to light after 10 d in the darkroom, the kitten could not pass an “open door” discrimination by visual cues alone but on the next day it could. On the third day in the light, it had recovered sufficient sight and confidence to jump and permit measurement of visual acuity on the jumping stand. The visual acuity achieved normal levels in both eyes ∼2 months later. [View online]

Important features of the apparatus and the description of the testing procedure can be appreciated by reference to the letters of Figure 2 that designate noteworthy features of the apparatus and the scale bar that provides a guide to its dimensions. Additional details of the apparatus in its various forms are provided in the original accounts (Mitchell et al., 1976, 1977). Kittens jumped from an open-ended rectangular box (A) on a platform (B) mounted upon two yoked laboratory jacks (C) that permitted continuous adjustment of the jumping distance to the two adjacent stimuli below. The stimuli were placed on the two (usually closed) trapdoors (D) that comprised the top of a large rectangular box (E) and were separated by a narrow divider that was 3 cm high (F). An incandescent lamp (L) provided illumination for the grating stimuli that had a luminance of 55 cd/m^2^. The jumping stand of Figure 2 displays the arrangement employed for older kittens of ∼4 months of age in order to emphasize the flexibility of the design. For the youngest kittens the laboratory jacks are placed directly on the floor so that the jumping platform can be lowered to match the height of the trapdoors for their initial training that requires that the kittens merely step onto the stimuli. As the kittens grow and their mobility matures, the height of the jumping stand can be increased very gradually as required by use of the jacks and in larger steps by placing rectangular boxes (G1, G2) of different sizes under the jacks. A mount (M) allowed for a GoPro camera to record the testing of some kittens at particular times in their recovery. The flexibility of its design, particularly with respect to the ability to change the observation (i.e., the jumping) distance in small steps, allows its use on kittens as young as 4 weeks. As they age, the jumping platform is raised gradually until at between 2 and 3 months of age, kittens are able to jump from 72 cm (as shown in Fig. 2) or higher.

The two adjacent trapdoors on which the stimuli were placed could separately be set as either open or closed with the latter as the situation in nearly all circumstances. The trapdoors were never used to punish incorrect choices as errors were met adequately by nothing more than denial of the rewards that accompany correct responses and fast repetition of the trial. As described below, the trapdoors were employed as an aid in the initial training of kittens on the jumping stand and also to document the very limited visual abilities of kittens that were for a time evident in the aftermath of a period of certain forms of early selected visual exposure.

## The Initial Training

It has been our experience that training should begin at between 4 and 5 weeks of age at which time they can first jump from the jumping platform albeit at a low height. For the first 10 trials of training of kittens, the jumping platform is set approximately flush with the height of the trapdoors. One trapdoor is opened with the positive stimulus, a vertical square-wave grating having a period of 40 mm, placed upon the closed trapdoor. The invariably correct response of the kitten to walk to the stimulus on the closed door is rewarded with immediate petting and a small piece of commercial cat food paté or small slivers from frozen chicken liver shavings placed on an ice-cream stick. After the first trial, the side of the closed trapdoor is changed for one or two trials. If thereafter the kitten walks freely to the grating, the height of the jumping platform is raised in small steps (1–5 cm) by use of the laboratory jacks (C) between trials depending upon the ease with which the kittens negotiates a step onto the grating. After 10 successful trials to the grating, the open trapdoor is closed to introduce the negative stimulus. The invariably correct initial trials (referred to as “open door” trials) together with the easy nature of the task in its initial stages provided an example of errorless discrimination that has been thought beneficial for training (Terrace, 1963). For the first few decades, a vertical grating was employed as the positive (rewarded) stimulus and a uniform gray stimulus of the same space-average luminance as the negative (unrewarded) stimulus. To reduce the probability that the animals could be influenced by aliasing (Hall and Mitchell, 1991) or possible small overall luminance differences between the stimuli, from around 1991 (Mitchell and Gingras, 1991) the negative stimulus was changed to a horizontal grating having the same period and average luminance as the (positive) vertical grating.

The animal is then further trained until it makes 10 consecutively correct responses to the vertical grating after which the period of the grating is reduced in logarithmic steps at a rate dictated by the animal's level of comfort as judged by the researcher. In response to any error, the kitten is denied the food reward and is required to immediately repeat the trial which it typically performs correctly. In addition to control of the speed with which the grating period is reduced, the researchers choose the appropriate jumping height to employ to improve the animal's level of comfort. Perhaps more importantly the researchers control the rate at which the grating period is reduced as the testing session proceeds. Over the years the procedure for measurement of visual acuity has been refined in order to permit repeatable measurements to be made in 50–90 trials. For longitudinal tests of visual acuity, we used large (19 × 19 cm) square-wave grating patterns with a gray border of the same luminance that was 3 cm wide.

## The Refinement of the Test Procedure

Whereas the jumping stand itself has changed very little since the original publication of the method nearly 5 decades ago, the test procedure has undergone considerable refinement in order to permit measurements of acuity on every animal and occasion. In essence the procedure has evolved to incorporate elements that resemble those employed on humans for measurements of thresholds in a clinical as opposed to a laboratory setting. For clinical testing of humans, it is important that the subject understands the task and is responding to the appropriate stimulus on the basis of visual cues alone and that their responses are not informed by extraneous factors such as guessing or memorization of the stimuli. Clinical testing involves a dialogue between the subject (patient) and the clinical practitioner so that the latter is assured that the subject understands the task and responds to appropriate aspects of the stimulus so as to enable accurate measurement of a visual threshold on each occasion. In contrast, laboratory tests of human visual thresholds require that the subject be physically isolated from the person administering the test with little or no dialogue between them once the test session starts. For laboratory testing the stimulus value on each trial is dictated by an algorithm such as a staircase procedure and the subject conveys their typically nonverbal response directly to a computer.

Over the years the procedures associated with use of the kitten jumping method have evolved so as to optimize performance of kittens throughout each test session by close monitoring and handling of the animal to allow measurements of thresholds on every kitten and occasion. As in the past and in common with human clinical test procedures, measurement of visual acuity for grating stimuli are accomplished by use of a descending method of limits with successive stimuli ordered in equal logarithmic steps as incorporated in the design of modern human clinical logmar acuity charts (Bailey and Lovie, 1976; Bailey and Lovie-Kitchin, 2013). But in contrast to the latter charts where the size of the letters on the lines of the acuity chart change size in 3 equal steps per octave, the period of the gratings employed on the jumping stand are spaced more closely with 10 or more equal steps to the octave (Murphy and Mitchell, 1987). Spacing of stimuli has been even finer (16 steps/octave) in other laboratories (Williams et al., 2015). The small step sizes that appear barely perceptible to a human reduce the likelihood that kittens abruptly adopt a new strategy between two adjacent grating sizes. The description of a typical test session that follows incorporates the changes made since the original description and includes the use as the negative stimulus, a horizontal grating having the same period as the paired vertical grating (the positive stimulus). However, the rewards for a correct response (petting and commercial cat food paté or small slivers of chicken liver) and consequences of an error (immediate repetition of the trial) remain the same as in the past. Other important aspects of the testing procedure are also unchanged since the original published description, notably the use of opaque hard contact lenses (from a choice of four different base curvatures) for monocular tests of acuity and use of a descending method of limits to measure acuity (defined as the highest spatial frequency for which the animal achieves criterion performance).

For a new kitten or for an animal following a period of deprivation, it is first necessary to establish a comfortable jumping distance for the jumping stand. As a guide we observe their spontaneous descents from the four boxes of different heights (Fig. 1) and then further refine the distance with rewarded jumps on the jumping stand itself. Following five training trials with one trapdoor open, the trapdoor is closed and the paired negative stimulus (a horizontal grating having the same period as the positive stimulus) is placed on the other trapdoor. The kitten is then trained until it makes 10 consecutively correct responses after which the period of the grating is reduced in logarithmic steps at a rate dictated by the animal's level of comfort as judged by the researchers. In addition to control of the speed with which the grating period is reduced, the researchers judge and tweak the jumping height by use of the laboratory jacks by close monitoring of the ease with which the kittens jump on each trial. For every session it is necessary to strike a balance between the number of trials required for each grating period, the number of such periods, and the total number of trials that kittens find comfortable on any day. Typically kittens require 3–5 sessions to learn the task and complete a sufficient number of trials to permit formal measurement of visual acuity. As expanded upon in the next section, the acuity on any session is defined as the grating having the highest spatial frequency for which the animal is correct on either five of five trials or after an error can either make five consecutively correct responses or a minimum of seven correct in the maximum of 10 trials provided for each grating period. These points are expanded upon in the next section.

## A Typical Test Session for a Trained Animal

A session begins with the same jumping distance as that employed previously unless there is reason to modify it on the basis of a subsequent delay or experiential intervention. As previously stated the method is a descending method of limits with a starting point (i.e., grating period) chosen on the basis of previous results in order to restrain the total number of trials in the test session. Just as a test of human letter acuity on an eye chart does not necessarily start with the largest letter size but with letters closer to the presumed threshold size based on prior information, test sessions on the jumping stand do not always begin with gratings having the largest period. For previously tested kittens the grating size at the beginning of the session may decrease in larger steps between trials and only a single trial provided for each period size. However within about 2 octaves of the presumed threshold based on previous results, the minimum number of trials for each grating period is increased to 2 and within an octave, to between 3 and 5. At any time after an error the animal must make five consecutively correct trials or at least seven correct in a maximum of 10 trials before the grating period is reduced. The threshold is defined as the smallest grating period (i.e., highest spatial frequency) for which the animal achieves this same criterion.

The conduct of a testing session is influenced by observations made by the researcher of the manner in which the animal makes its decision and the degree to which its behavior appears to be dictated exclusively by visual cues. For example, it has been known for many years that in two-alternative forced-choice discriminations, animals can adopt side preferences or alternation response patterns. To forestall these unwanted behavior patterns, the pseudorandom order presentation developed by Gellerman (1933) is used to avoid more than two successive repeated presentations on one side or successive alternation sequences. Occasionally additional adjustments are made on the basis of the researcher's observations of the animals behavior such as a hesitation to look or jump to one side, the speed with which they make their choices and signs of hesitation such as prolonged looking at other objects near the jumping stand that can occur near their threshold. In order to forestall the development of a side preference a second researcher sits on the other side of the jumping stand, an arrangement evident in each of the movie clips that follow; while one researcher sets the gratings for the next trial and records the results of each trial, the second researcher provides the rewards and returns the kitten to the jumping platform for each trial. The side of presentation of the positive stimulus can be modified for a few trials if it is judged that the animal's choices are not entirely guided by visual cues alone. Most importantly, observations of the animal's behavior during the test sessions dictates important parameters such as the number of trials provided for each grating period and the cessation of the test session. One important observation is whether the kitten appears to look at one or both stimuli before commitment to a choice. At the beginning of the test session, the kitten may make its choice on the basis of a quick look at just one of the stimuli but as the session progresses it looks at both stimuli and sometimes more than once before it jumps. Characteristically, kittens change from the first to the second mode of looking as the trial progresses and the task becomes progressively more difficult as the animals take longer and appear more hesitant to make their choice. Within one or two gratings from threshold kittens, the latency before a jump may last several minutes, and they may also try to back off the jumping platform in order to avoid a decision. However, at a certain grating size they appear to quit making a choice on the basis of visual cues and instead turn their head to look at the experimenter or other objects in the room and then jump repeatedly to the same side without any hesitation. As animals may exhibit signs of distress such as crying at this point, we cease the session after three consecutive errors and accept the previous grating size as the threshold. The varied pattern of individual behavior exhibited by kittens during a test session are reminiscent of the various cues that guide judgements by observers of the side of the grating during preferential looking sessions on human infants (Teller, 1979). Over many years we remain satisfied that measures of acuity obtained with the jumping stand provides an accurate estimate in angular terms (i.e., spatial frequency) because a change in the jumping distance results in a change of the threshold in terms of the period of the grating but not in terms of its spatial frequency.

Despite other changes made in the last few decades, the procedure with respect to the duration of the session and the number of trials remains largely unchanged. A typical session begins with gratings having a period of 40 mm. On successive trials unless the animal makes an error, the period is reduced in logarithmic steps with at first 4, 5, and then 10 steps per octave. After an error the animal is required to correct its response and then make either five consecutively correct jumps or a minimum of seven correct responses in 10 trials before the grating period is reduced once more. Once trained, a typical session requires from 50 to 90 trials and lasts ∼25 min. On most sessions kittens eat a small amount of food dispensed on a wooden ice-cream stick accompanied by petting on every trial but petting alone is sufficient once they become satiated with food. It is never necessary to food deprive the animals prior to a test session.

## An Additional Role for the Trapdoors

In addition to their employment during initial training, the trapdoors were also used as an index of the very limited visual abilities of one or both eyes of kittens that followed certain periods of selected early visual exposure such as darkness (Timney et al., 1978), as well as monocular or binocular visual deprivation (Mitchell, 1988). If it was apparent that an animal was able to step or even jump onto the closed trapdoor without touching it first, the animal was judged as having attained sufficient visual ability to make an “open door” discrimination, an ability that might reflect very rudimentary form vision or even just the ability to detect a luminance difference between the open and the closed trapdoors. Such rudimentary levels of form vision could be thought of as analogous to designations such as “light perception” or “count fingers” at a specified close distance to define levels of form vision in humans that are insufficient to permit identification of the largest letters on an eye chart even at very close range (Bailey et al., 2012; Bailey and Lovie-Kitchin, 2013).

The following three short video clips illustrate the procedure and the characteristic patterns of behavior exhibited by kittens with different visual abilities in one or both eyes (Movies 1–3).

## Discussion

### Importance of a high level of commitment to signal choice

The development of the jumping stand was founded upon the spontaneous and eager attempts of kittens to descend from an elevated surface. It was possible to shape this willingness and urgency to jump to signal the position of a vertical grating and ultimately the grating having the highest spatial frequency that controlled this behavior. The necessity to maximize the jumping distance from an early age so as to increase the spatial frequency of the grating stimuli to threshold levels of visibility ensured a continued high level of vigilance and commitment prior to each jump. Because incorrect responses incur expenditures in terms of vigilance and the metabolic demands of subsequent jumps, the level of motivation remained high throughout each testing session. The assertion of a high level of motivation provided by the willingness and urgency of kittens to signal the positive stimulus by a jump toward it receives additional support from prior results reported on rats by Lashley (1930) on his jumping stand as compared with prior results obtained from use of conventional 2AFC runways. With Lashley's jumping stand, the rats had to signal their choice by a horizontal jump from an elevated platform toward one of two doors; correct jumps to the stimulus on one door were rewarded by the door opening and food, while errors were met by a closed door so that the rat fell to the floor and was not rewarded with food. Recently, young rodents in two-choice discriminations have been shown to exhibit superior visual acuity when tested on a two-choice water maze in which they are required to swim to a hidden platform beneath the positive stimulus (Prusky et al., 2000a,b; Prusky and Douglas, 2003). As there was no platform beneath the negative stimulus, after an error the animal had to swim the additional distance to the positive stimulus to find the safe platform. In support of the contention of the willingness and urgency of animals to signal a visual choice by jumping as opposed to slower modes of response such as walking or swimming are a growing number of reports of the use of modified designs of the jumping stand for measurements of the visual acuity of species as diverse as *Dasyurus hallucatus*, the carnivorous Australian native cat (Harman et al., 1986), and chickens (Over and Moore, 1981).

### Examples of use of the kitten jumping stand

The motivation for the development of the jumping stand was to permit documentation of the immediate consequences of periods of early selected visual exposure for visual spatial thresholds such as visual acuity and contrast sensitivity. Many studies from my laboratory have confirmed that the fast electrophysiological changes observed in the visual cortex of selectively visually deprived kittens are accompanied by equally rapid and profound changes in spatial visual thresholds. During the early years of use of the jumping stand, we were conscious of the possible use by kittens of cues linked to the behavior of the humans that handled and tested them. At the outset we concentrated upon experiential and other manipulations for which the behavioral consequences and the speed with which they occurred were either not known or could not be predicted in any detail. A case in point were the consequences of termination of periods of reverse occlusion. Although the behavioral events during the latter conformed to expectations based on the outcome of prior electrophysiological studies (Movshon, 1976), the changes in vision that occurred in each eye after the period of reverse occlusion ended were quite unexpected. Importantly, the visual acuity recovered by the originally deprived eye during the period of reverse occlusion declined very rapidly afterward (Murphy and Mitchell, 1987). A possible anatomical explanation for this visual loss in terms of changes in the morphology of geniculocortical afferents was not provided until much later (Antonini et al., 1998). A second example followed from consideration of the possible consequences for the development of vision of infant humans of brief episodes of accidental abnormal visual exposure during early postnatal life as could arise from brief recurring corneal infections or trauma in one eye. To examine this possibility, my laboratory exploited the presence of a now 50-year-old darkroom colony room (Mitchell, 2013) to provide kittens with mixed visual exposure each day. For kittens that were otherwise kept with their mother in the darkroom so as to restrict their daily visual exposure to 7 h, it was shown that just 2 h of daily typical binocular visual experience could offset 5 h of exclusively monocular exposure to allow both the development of normal visual acuity in each eye (Mitchell et al., 2006) and normal ocular dominance domains in the visual cortex (Schwarzkopf et al., 2007). Almost identical results were obtained in parallel independent studies conducted on monkeys (Sakai et al., 2006).

A second more recent example was provided by the unexpected consequence of a short 10 d period of total darkness (Duffy and Mitchell, 2013; Mitchell et al., 2016) or binocular retinal silencing with tetrodotoxin (Fong et al., 2016) for kittens that had received a prior period of monocular deprivation. When imposed at certain ages, either manipulation could promote fast recovery from the prior period of monocular deprivation to allow for fast attainment of normal visual acuity in both eyes. Finally, a long study has employed the jumping stand to document the consequences for visual acuity and other visual functions of ablation of the cortical areas 17 and 18 in either kittens or adult cats (Mitchell, 2002).

### The importance of the animal model

From the outset of my work on animals in the early 1970s, I have been able to use cats to model human developmental visual disorders and build upon the legion of anatomical and electrophysiological studies that have been conducted on this species until comparatively recently. As frequently mentioned, cats provide an excellent animal model to study the development of normal and abnormal vision because of the similarity of the organization and plasticity of their visual pathways to those of primates including humans (Mitchell and Sengpiel, 2018). The far greater ease of study of cats over nonhuman primates from the perspectives of availability, litter size, and cost has resulted in windfall of information concerning the anatomical, cellular, and physiological development of the visual pathways that subserve vision. From the perspective of behavioral studies, another important benefit of cats is that they are a highly domesticated species. Allied with their willingness to perform on the jumping stand, the extensive handling they receive during their critical period of socialization from 2 to 7 weeks of age (Karsh and Turner, 1988; Stella and Buffington, 2014) has facilitated the parallel collection of data on the development of both monocular and binocular visual functions. A likely additional contributor to the success of the method is the significant level of commitment and vigilance required by the kitten to signal their response by a directed jump allied to the concomitant cost of an error that requires immediate repetition of the trial. When initiated early at between 4 and 5 weeks of age kittens are comparatively easy to train and if tested regularly they remain very tractable and friendly into early adulthood at which time the majority are homed to members of the university community.

The last 50 years has seen the accumulation of a considerable database on the anatomical, cellular, and physiological development of the kitten visual pathways as well as on the visual functions they subserve. Moreover, an equally large database has been established on the consequences of periods of different forms of early visual exposure on kittens that has been driven in part by the desire to reach an understanding of the underpinnings of developmental visual disorders such as amblyopia. However, in parallel with the accumulation of this valuable information, increased pressure and barriers have been applied or erected from various sources to motivate replacement of kittens by rodents and particularly mice for developmental studies of vision (Mitchell and Sengpiel, 2018). The work conducted to date on kittens provides an invaluable body of information to guide future studies on this species of experiential manipulations (or other methods) for treatment of amblyopia in humans. At present it is still possible to conduct research directly related to amblyopia on a number of species from nonhuman primates, cats, to rodents. A detailed account of the efficacy of the common animal models of amblyopia with respect to their ability to provide a foundation for human intervention was provided by a panel of the Lasker/IRRF Initiative for Innovation in Vision Science (Amblyopia: Challenges and Opportunities) in March 2017. The conclusions of this panel (Mitchell and Sengpiel, 2018) include detailed recommendations for future study of the different animal models and their respective value for an eventual understanding of the origins and treatment of amblyopia. However the ability to proceed in an orderly fashion as advocated by this panel has been impacted by progressively growing restrictions from various sources on the use of particular animal species coupled with pressure and even directives for the exclusive use of rodent animal models. To restrict the choice of animal models to the use of rodents to guide such studies is at the least misguided and brings to mind the century-old story of the person who drops a key at night in the middle of a residential street but chooses not to look for it there but instead under a lamp post because of the better light there.

## Synthesis

Reviewing Editor: Christophe Bernard, INSERM &Institut de Neurosciences des Systèmes Decisions are customarily a result of the Reviewing Editor and the peer reviewers coming together and discussing their recommendations until a consensus is reached. When revisions are invited, a fact-based synthesis statement explaining their decision and outlining what is needed to prepare a revision will be listed below. The following reviewer(s) agreed to reveal their identity: Mark Bear.

Dear Donald,

I am so sorry about the delay, but one reviewer turned out to be a no show.

There are minor things recommended by one reviewer

best

This paper is a gem and should be required reading for all those who use or seek to use behavior to assess visual capabilities in animals-particularly in animal models of human visual disorders, such as amblyopia.

The author provides a historical overview of the testing procedures and refinements made to the kitten jumping stand task to facilitate fast capture of visual thresholds of all subjects (kittens) under varying conditions. The paper gives a detailed description of the task, procedures used for training and testing subjects, as well as pitfalls and strategies for overcoming individual differences in performance/motivation. Figures, in the form of photographs, accompany the manuscript to ensure that both the experimental setup and procedures can be replicated by interested researchers. The detailed description of testing procedures such as descending method of limits is an important reminder to visual neuroscientists of how testing in the lab can best be performed so that they model procedures used for visual testing in the clinical setting. Additionally, it is through the author's introductory descriptions that the reader comes to know the developmental history of the task and that it was designed to emulate features of basic tests of human spatial vision as applied in the clinic rather than those in a laboratory setting. This is a particularly important distinction.

Suggestions:

1. The article is clearly written and the length appropriate. Although this is not a criticism, if permissible it would be of interest to provide a link in which the reader could view actual videos of behavioral trials demonstrating subject successes/failure in the task. This may require video masking or done through animation.

2. I think a few tweaks would broaden the appeal of the paper. For example, the title is accurate but could be more engaging. Perhaps better would be something pithy, like "My 50-year odyssey to develop behavioral methods that let me see what kittens see". Additionally, and I think importantly, the abstract should start with a sentence stating how important animal models are to understand the development and plasticity of visual functions, and why kittens, in particular. Historically, studies in kittens paved the way for understanding the general principles that apply to all species with good binocular vision (and even some that don't, like the mouse). We still have much to learn from kittens, as they share the key properties of visual system organization and plasticity with primates (e.g., laminated LGN, OD and orientation columns), but are far easier to study than primates (availability, expense, litter sizes, domestication) and have a very substantial database with which results can be compared. The sad truth is that an entire generation of upcoming neuroscientists are so mouse-centric that they've lost sight of what they are actually trying to model in animals, particularly when it comes to conditions such as amblyopia.
